# Supplementary material for: The Barley Heavy Metal Associated Isoprenylated Plant Protein HvFP1 Is Involved in a Crosstalk between the Leaf Development and Abscisic Acid-Related Drought Stress Responses
Source: Plants (Basel). 2022 Oct 26;11(21):2851. doi: 10.3390/plants11212851 (PMC9657915; doi:10.3390/plants11212851)
Supplement: Supplementary file 1 [file plants-11-02851-s001.zip › plants-1953690-supplementary.pdf]

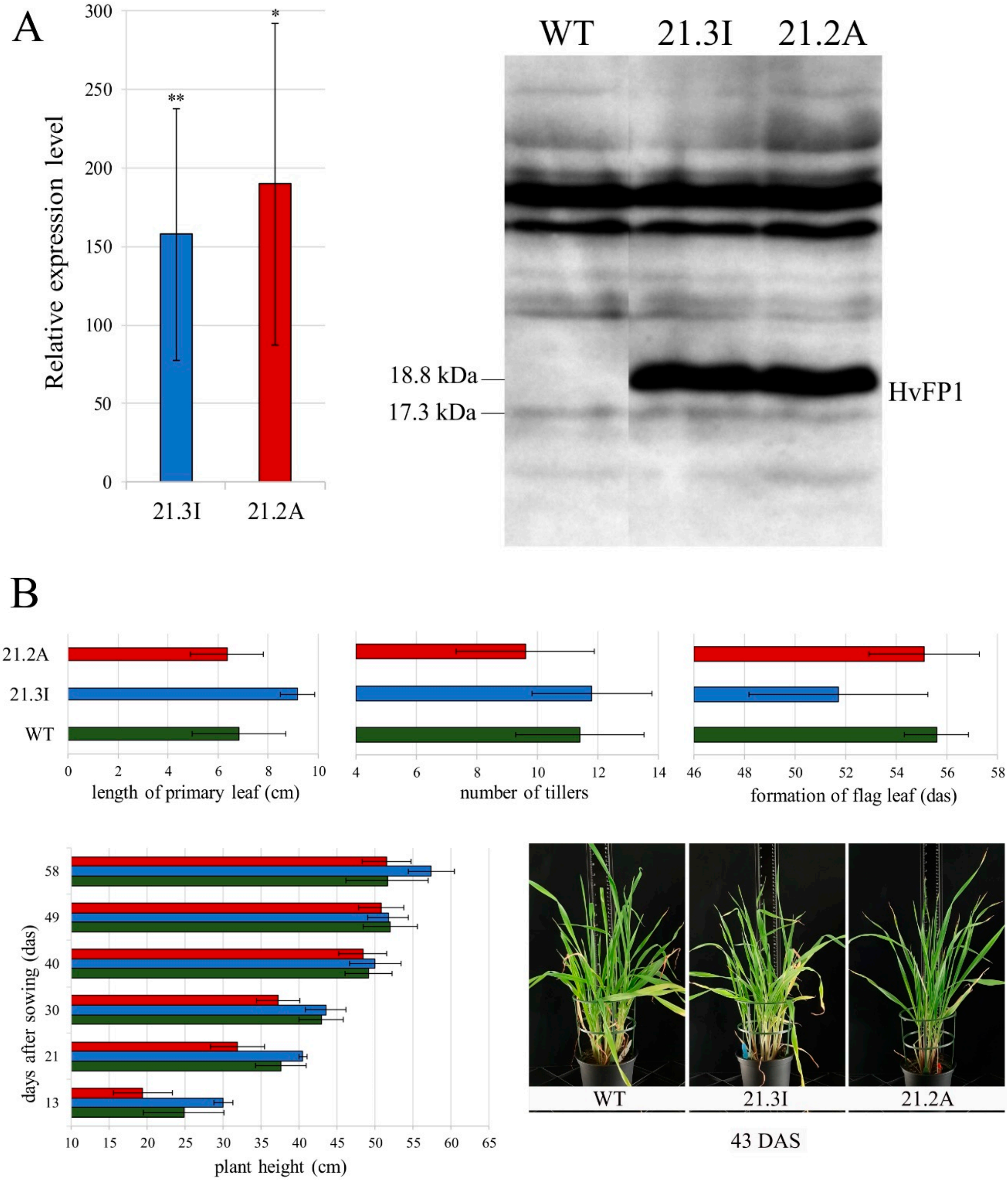

Figure S1. Genotypic and Phenotypic characterization of *HvFP1* OE lines **(A)** Left: The relative transcript level of *HvFP1* in barley overexpression (OE) transgenic lines 21.3I and 21.2A in comparison to WT samples on 11th day after sowing (DAS). Mean relative expression levels of at least three samples, standard errors and *p*-values were determined by REST-384 © 2006 (Relative Expression Software Tool - 384, version 2; [14], Qiagen GmbH, Hilden, Germany) and normalized against *HvPP2A*, *HvActin* and *HvGCN5*. The statistical significance between OE samples in comparison to WT samples is indicated by asterisks: $p < 0.05$ (*), $p < 0.01$ (**). Right: Detection of HvFP1 in WT and both OE lines in a western blot using purified polyclonal anti-HvFP1 antibodies. Predicted protein size for WT HvFP1 is 17.3 kDa and for OE HvFP1 with the strep-tag is 18.8 kDa.

(B) Phenotypic analysis of WT and *HvFP1* OE lines in terms of primary leaf length (in cm), number of tillers, flag leaf formation (on various DAS) and plant height (on various DAS). A photo of barley plants on 43rd DAS shows no obvious difference during plant cultivation.

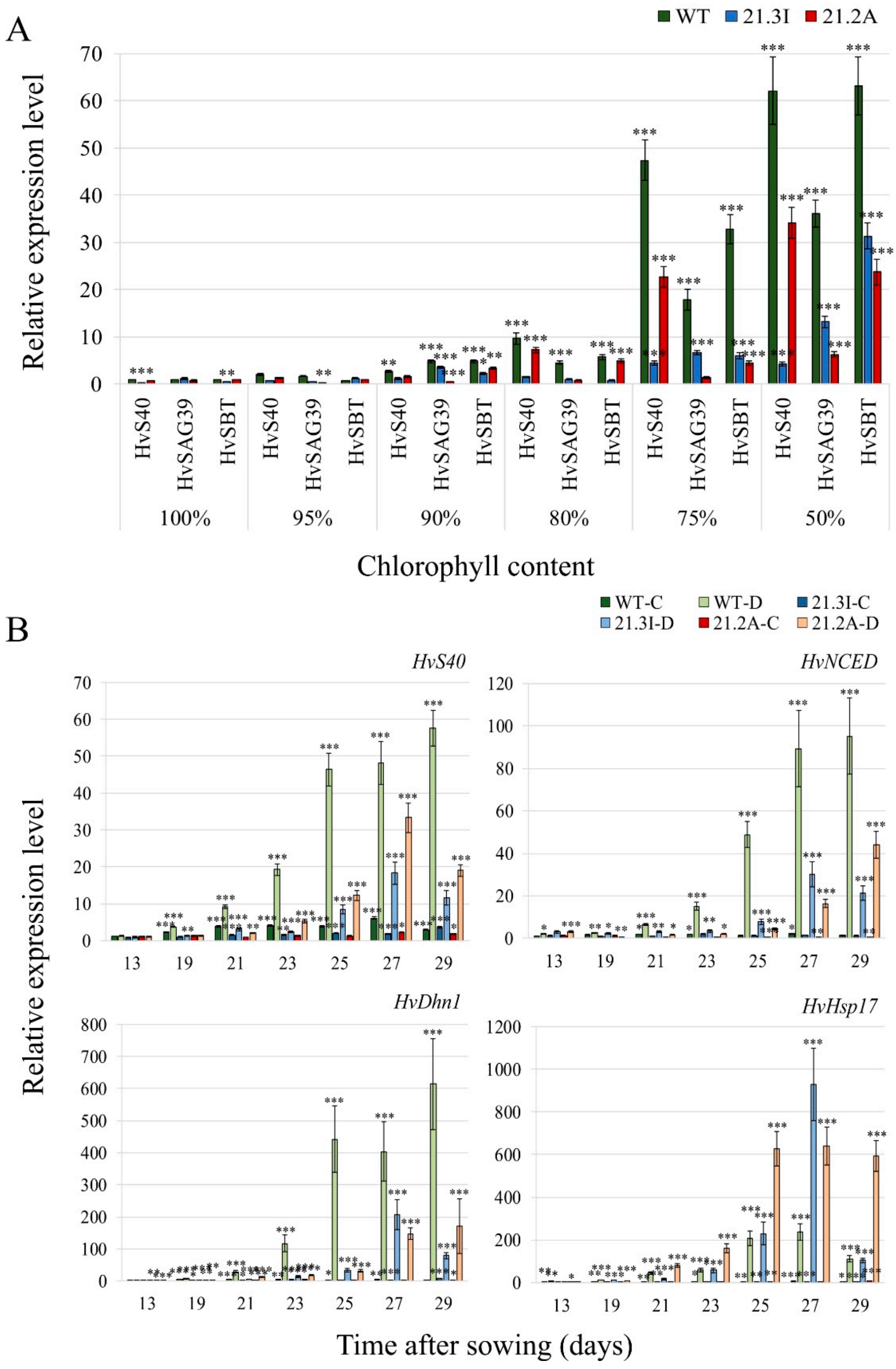

Figure S2. The relative transcript level of specific genes during developmental- and drought induced- leaf senescence: **(A)** compared with samples of WT on the 13th DAS (set as 1), at various stages of developmental leaf senescence as defined by the chlorophyll content of WT primary leaves, and **(B)** at different time points of drought induced senescence, compared with samples of WT on the 11th DAS (set as 1). Mean relative expression level of three independent biological replicates, standard errors and *p*-values were determined by REST-384 © 2006 (Relative Expression Software Tool - 384, version 2; [14], Qiagen GmbH, Hilden, Germany) and normalized against *HvPP2A*, *HvActin* and *HvGCN5*. Statistically significant differences between samples of **(A)** WT line at various developmental stages and OE lines on corresponding days in comparison to WT samples of 13th DAS, and **(B)** control and drought treatments of all lines on various DAS in comparison to WT control samples of 11th DAS, are indicated by asterisks: p <0.05 (*), p <0.01 (**), p <0.001 (***).

Table S1: Differentially expressed genes in *HvFP1* OE line. Genes are sorted according to log_2FoldChange (log_2FC) in mature leaves. Gene ids, gene names and log_2FC are provided.

Gene ID	Gene description	log2FC Mature	log2FC Senescence
HORVU.MOREX.r2.3HG0209440	40S ribosomal protein	10.09891772	6.33140765
HORVU.MOREX.r2.3HG0224010	DNL-type zinc finger	9.341489827	9.040781066
HORVU.MOREX.r2.1HG0051090	Kinesin heavy chain	9.239889174	5.604245085
HORVU.MOREX.r2.5HG0376190	1-phosphatidylinositol 4,5-bisphosphate phosphodiesterase epsilon-1	9.169492147	9.220986694
HORVU.MOREX.r2.5HG0395730	Phytochromobilin:ferredoxin oxidoreductase, chloroplastic	8.977770186	9.267850002
HORVU.MOREX.r2.5HG0351050	Phosphoenolpyruvate carboxylase	8.905694944	8.287028057
HORVU.MOREX.r2.6HG0520340	Tetratricopeptide repeat (TPR)-like superfamily protein	8.76554996	8.403128446
HORVU.MOREX.r2.5HG0433760	Pyridoxal-5'-phosphate-dependent enzyme family protein	8.657540925	8.797713735
HORVU.MOREX.r2.2HG0130560	Proteasome subunit beta type (type 3-A; subunit C-1)	8.417070723	7.728959767
HORVU.MOREX.r2.1HG0012690	Dynein assembly factor 1, axonemal	8.390796608	8.460856648
HORVU.MOREX.r2.1HG0012700	HAT transposon superfamily	8.298905518	8.448098918
HORVU.MOREX.r2.4HG0305130	Auxin response factor 10	8.150846777	7.798846489
HORVU.MOREX.r2.7HG0578290	DNA ligase	7.989434019	5.810782985
HORVU.MOREX.r2.1HG0023890	Phospho-N-acetylmuramoyl-pentapeptide-transferase	7.918639431	8.34873524
HORVU.MOREX.r2.5HG0397670	Transposon protein, putative, CACTA, En/Spm sub-class	7.887295637	6.975176729
HORVU.MOREX.r2.6HG0520330	binding protein	7.686417974	7.93531421

HORVU.MOREX.r2.6HG0455790	Histone H3	7.633630144	7.555058833
HORVU.MOREX.r2.3HG0259090	Muscle calcium channel subunit alpha-1	7.5319633	
HORVU.MOREX.r2.3HG0249140	Elongation factor Ts	7.372007293	7.171357204
HORVU.MOREX.r2.3HG0212380	fibronectin type III domain protein (DUF1423)	7.233525244	7.90076122
HORVU.MOREX.r2.5HG0371730	Transposon protein, putative, Pong sub-class	7.222743431	6.845235332
HORVU.MOREX.r2.4HG0320800	Chaperone DnaJ-domain superfamily protein	7.15711592	7.028714062
HORVU.MOREX.r2.6HG0478560	callose synthase 1	7.049522471	7.124573949
HORVU.MOREX.r2.7HG0608000	DNA polymerase alpha subunit B	7.017177497	8.846737482
HORVU.MOREX.r2.3HG0240610	Transposon protein, putative, CACTA, En/Spm sub-class	6.947747202	6.893257629
HORVU.MOREX.r2.4HG0316380	FAR1-related sequence 5	6.848703004	8.675910728
HORVU.MOREX.r2.2HG0085430	glycosyl hydrolase family 10 protein / carbohydrate-binding domain-containing protein	6.840970914	7.148889865
HORVU.MOREX.r2.5HG0445070	DEAD-box ATP-dependent RNA helicase 52A	6.8113738	4.682553834
HORVU.MOREX.r2.7HG0610720	Werner Syndrome-like exonuclease	6.602603503	
HORVU.MOREX.r2.3HG0212390	Muscarinic acetylcholine receptor M3	6.569672214	6.342852564
HORVU.MOREX.r2.5HG0351060	Rp1-like protein	6.561642319	
HORVU.MOREX.r2.2HG0164100	SH3 domain-binding protein 1, putative	6.285956788	7.11657807
HORVU.MOREX.r2.5HG0399970	Chlororespiratory reduction31; NAD(P)H-quinone oxidoreductase subunit S, chloroplastic	6.169954116	8.98004611
HORVU.MOREX.r2.2HG0084340	La-related protein 7	6.075934077	

HORVU.MOREX.r2.4HG0309140	Microtubule-associated protein RP/EB family member; Protein ATEB1 homolog 2;	5.919337871	7.476759222
HORVU.MOREX.r2.7HG0602190	DHHC-type zinc finger family protein	5.756455769	7.53874664
HORVU.MOREX.r2.1HG0017340	Dynamin-1	5.744607717	
HORVU.MOREX.r2.5HG0352380	Myb/SANT-like DNA-binding domain protein	5.519518211	7.548061139
HORVU.MOREX.r2.2HG0086370	Heavy metal-associated domain containing protein (HvFP1), expressed; AtHIPP27	5.428575674	
HORVU.MOREX.r2.7HG0578300	Glutamate receptor	5.073129474	7.644318911
HORVU.MOREX.r2.5HG0392660	XH/XS domain-containing protein	4.971050561	4.854054237
HORVU.MOREX.r2.7HG0578310	Sentrin-specific protease 1	4.838731948	6.791997313
HORVU.MOREX.r2.2HG0171210	Alpha/beta-Hydrolases superfamily protein	4.737839115	
HORVU.MOREX.r2.7HG0585880	Zinc finger CCCH domain-containing protein 12 (-ZFN-like 2) (OsC3H12)	4.587184641	4.506394085
HORVU.MOREX.r2.7HG0602170	Regulator of chromosome condensation (RCC1) family with FYVE zinc finger domain-containing protein	4.574567919	4.795465873
HORVU.MOREX.r2.7HG0531390	HAT dimerisation domain-containing protein-like	4.520412972	4.056622937
HORVU.MOREX.r2.7HG0553540	SUMO-activating enzyme 2	4.230148952	4.84788024
HORVU.MOREX.r2.1HG0006800	RNA polymerase Rpb1, domain 2 family protein	4.130422985	5.544955014
HORVU.MOREX.r2.3HG0207920	photosystem I reaction center subunit PSI-N, chloroplast, putative (PSAN)	4.053132782	4.827737438
HORVU.MOREX.r2.4HG0298030	Laccase-22	3.94852614	5.797146394
HORVU.MOREX.r2.7HG0531400	Leucine-rich repeat receptor-like protein kinase family protein	3.7897057	

HORVU.MOREX.r2.4HG0324960	Ubiquitin; Ubiquitin-60S ribosomal protein L40-2	3.7180326	
HORVU.MOREX.r2.6HG0489720	Wall-associated receptor kinase 1	3.669961509	4.222181232
HORVU.MOREX.r2.4HG0302280	Sterol 3-beta-glucosyltransferase	3.577997457	
HORVU.MOREX.r2.5HG0378770	Acyl-[acyl-carrier-protein] desaturase; Stearoyl-ACP desaturase 5	3.534739319	4.493157475
HORVU.MOREX.r2.2HG0174650	Ubiquitin-conjugating enzyme, E2 23	3.512174889	
HORVU.MOREX.r2.4HG0325150	Rho GTPase-activating protein; Protein ROP1 ENHANCER 1 (REN1)	3.421371332	
HORVU.MOREX.r2.3HG0223050	protein serine/threonine kinase	3.29157322	4.225990947
HORVU.MOREX.r2.4HG0299360	Protein kinase; Putative cysteine-rich receptor-like protein kinase 20	3.228197529	2.687493532
HORVU.MOREX.r2.5HG0396600	vaculolar sorting receptor 3	3.20863673	
HORVU.MOREX.r2.1HG0068040	CheY-like two-component responsive regulator family protein	3.190203898	2.987197457
HORVU.MOREX.r2.2HG0113950	Beta-amylase; 1,4-alpha-D-glucan maltohydrolase	3.071919265	4.714970172
HORVU.MOREX.r2.4HG0314620	ATP-dependent helicase HRQ1; ATP-dependent DNA helicase MER3 homolog	2.678803432	2.960664848
HORVU.MOREX.r2.2HG0105250	Dihydroflavonol-4-reductase; Dihydrokaempferol 4-reductase	2.633406305	
HORVU.MOREX.r2.5HG0404540	NADH-quinone oxidoreductase subunit C	2.498024595	
HORVU.MOREX.r2.7HG0578320	Nitrate transporter 1.1; Protein NRT1/ PTR FAMILY 6.4 (NPF 6.4)	2.405650631	
HORVU.MOREX.r2.3HG0183540	Disease resistance protein RPM1 (Resistance to Peronospora parasitica protein 1)	2.231054822	
HORVU.MOREX.r2.4HG0304020	FRS (FAR1 Related Sequences) transcription factor family	2.135575436	
HORVU.MOREX.r2.6HG0483180	Protein FAR1-RELATED SEQUENCE 1; FAR1-RELATED SEQUENCE 5		3.986328805
HORVU.MOREX.r2.5HG0362360	Protein FAR1-RELATED SEQUENCE 5		3.850814209

HORVU.MOREX.r2.2HG0134440	Zinc finger CCCH domain-containing protein 43 (OsC3H43), Myb/SANT-like DNA-binding domain protein	3.795834218
HORVU.MOREX.r2.7HG0584560	ARIA-interacting double AP2 domain protein	3.623348401
HORVU.MOREX.r2.UnG0634460	NAD(P)H-quinone oxidoreductase subunit 3, chloroplastic	3.444272039
HORVU.MOREX.r2.1HG0022230	MAR-binding filament-like protein 1-1	2.933144765
HORVU.MOREX.r2.6HG0460140	Endoglucanase 11	3.413180116
HORVU.MOREX.r2.4HG0302250	Acyl-CoA-binding domain-containing protein 4	4.100930099
HORVU.MOREX.r2.2HG0169350	Probable staphylococcal-like nuclease CAN3; Ca(2+)-dependent nuclease 3	4.559752321
HORVU.MOREX.r2.6HG0483610	tRNA pseudouridine synthase D	5.484159666
HORVU.MOREX.r2.5HG0405890	tRNA (guanine-N(7)-)-methyltransferase	4.331513255
HORVU.MOREX.r2.3HG0183280	Derlin-2.1	2.984953992
HORVU.MOREX.r2.5HG0362860	Inter-alpha-trypsin inhibitor heavy chain H3	7.597741335
HORVU.MOREX.r2.7HG0529200	MLO-like protein 1	4.684755781
HORVU.MOREX.r2.2HG0134450	Tripartite terminase subunit 1	3.681578562
HORVU.MOREX.r2.3HG0240540	modifier of snc1,4	2.851320406
HORVU.MOREX.r2.2HG0174540	Disease resistance RPP13-like protein 4 (or ZAR1)	2.562421756
HORVU.MOREX.r2.7HG0593770	Receptor kinase-like protein Xa21	4.699735273
HORVU.MOREX.r2.6HG0514330	Protein kinase family protein	3.872066631
HORVU.MOREX.r2.6HG0514320	Cysteine-rich receptor-like protein kinase 24	3.538964616

HORVU.MOREX.r2.4HG0318530	calmodulin 1	3.065218435
HORVU.MOREX.r2.1HG0068030	Transposon protein Pong sub-class	6.483000943
HORVU.MOREX.r2.7HG0578120	HAT family dimerisation domain containing protein	6.435725732
HORVU.MOREX.r2.7HG0534230	Transposon protein, putative, Mutator sub-class	3.994039843
HORVU.MOREX.r2.1HG0028670	Transposon protein, putative, CACTA, En/Spm sub-class, expressed	3.878676044
HORVU.MOREX.r2.6HG0471730	Retrovirus-related Pol polyprotein from transposon TNT 1-94	6.308430208
HORVU.MOREX.r2.2HG0111810	Transposase	3.718253356
HORVU.MOREX.r2.3HG0227220	Auxilin-like protein 1	3.800581988
HORVU.MOREX.r2.2HG0125080	Protein GRIP	2.781183841
HORVU.MOREX.r2.3HG0211130	Kinesin-like protein KIN-14T	3.48602842
HORVU.MOREX.r2.1HG0077060	Structural maintenance of chromosomes protein 3	3.477101684
HORVU.MOREX.r2.7HG0576930	Structural maintenance of chromosomes protein 1	3.091649829
HORVU.MOREX.r2.3HG0223070	Animal RPA1 domain protein	4.15729484
HORVU.MOREX.r2.3HG0223060	Replication protein A 70 kDa DNA-binding subunit	3.824092839
HORVU.MOREX.r2.4HG0290210	Fanconi anemia group M protein; ATP-dependent RNA helicase FANCM (or MPH1)	2.910316712
HORVU.MOREX.r2.7HG0572880	Helicase-like protein	3.237593751
HORVU.MOREX.r2.1HG0075950	DNA topoisomerase 2	5.951933713
HORVU.MOREX.r2.7HG0595240	Cell division cycle and apoptosis regulator protein 1	2.590300024

HORVU.MOREX.r2.5HG0407770	Helicase SEN1; tRNA-splicing endonuclease positive effector		2.492422916
HORVU.MOREX.r2.2HG0166400	Sentrin-specific protease		6.408309759
HORVU.MOREX.r2.2HG0115750	Tetratricopeptide repeat (TPR)-like superfamily protein		6.437306691
HORVU.MOREX.r2.1HG0032640	F-box protein		3.432762353
HORVU.MOREX.r2.4HG0338220	Dentin sialophosphoprotein-related, putative isoform 1		2.757074692
HORVU.MOREX.r2.6HG0461250	F-box family protein	−3.125992813	−6.292576622
HORVU.MOREX.r2.4HG0277340	Protein DETOXIFICATION	−2.646252216	−3.29600419
HORVU.MOREX.r2.5HG0436300	RNA polymerase II transcription mediator		−7.399913151
HORVU.MOREX.r2.6HG0471520	Histone H3		−6.673454006
HORVU.MOREX.r2.UnG0635260	Mediator of RNA polymerase II transcription subunit 12		−6.205632578
HORVU.MOREX.r2.4HG0345940	rRNA N-glycosidase		−5.481723283
HORVU.MOREX.r2.UnG0634520	Senescence-associated protein, putative		−5.273073872
HORVU.MOREX.r2.UnG0635170	FGGY family of carbohydrate kinase		−4.428450273
HORVU.MOREX.r2.UnG0632300	Senescence-associated protein, putative		−4.345757984
HORVU.MOREX.r2.7HG0607010	Ras-related protein RHN1		−3.847279247
HORVU.MOREX.r2.3HG0242690	Peroxidase 2		−3.262583911
HORVU.MOREX.r2.UnG0628140	Gibberellin 3-beta-dioxygenase 1		−2.818349381

Table S2: *Cis*-acting regulatory elements in possible *HvFP1* promoter region, 2 kb sequence upstream of 5'-UTR of *HvFP1* gene

***Cis*-acting regulatory element**	**Function**
ABRE, ABRE3a, ABRE4	abscisic acid responsiveness
CGTCA-motif	MeJA-responsiveness
TGACG-motif	MeJA-responsiveness
TATC-box	gibberellin-responsiveness
TGA-element	auxin-responsive element
CCAAT-box	MYBHv1 binding site for stress and defense responses
ARE	anaerobic induction
GC-motif	anoxic specific inducibility
DRE core	cold and dehydration responsiveness
LTR	low-temperature responsiveness
ACE	light responsiveness
AE-box	light response
ATC-motif	light responsiveness
ATCT-motif	light responsiveness
G-Box	light responsiveness
Sp1	light responsive element
CAT-box	meristem expression
CCGTCC-box	meristem activation
O2-site	zein metabolism regulation
RY-element	seed-specific regulation
TATA-box	core promoter element around -30 of transcription start
CAAT-box	cis-acting element in promoter and enhancer regions
MYB recognition site	MYB binding site
MYC	MYC binding site
W box	WRKY binding site

Table S3: Primers used in qRT-PCR. Lists of gene IDs, gene names, primer names, primer sequences and amplicon sizes are provided.

Gene IDs	Gene name	Primer name	Primer sequence	Amplicon size
HORVU4Hr1G074680	Serine/threonine protein phosphatase 2A	HvPP2A_fw	5'- CACCATTTCTCAGCTTGTATTG - 3'	100 bp
		HvPP2A_rev	5'- CACCCCTTTGTTATTGTTTGTTG - 3'	
HORVU1Hr1G002840	Actin 7	HvActin_fw	5'- GGAAATGGCTGACGGTGAGGAC - 3'	122 bp
		HvActin_rev	5'- GGCGACCAACTATGCTAGGGAAAAC - 3'	
HORVU1Hr1G034070	Histone acetyltransferase GCN5	HvGCN5_fw	5'- CAGGCCGCGTCAACCAAGAAC - 3'	96 bp
		HvGCN5_rev	5'- GGACGGCATAACAAGCAAGTCAG - 3'	
HORVU1Hr1G081240	Protein of unknown function, DUF584	HvS40_fw	5'-CGACGGCGACGTCCGATGTA-3'	137 bp
		HvS40_rev	5'-CTTTGAGCGTCCTCCCTTTGC-3'	
HORVU5Hr1G008050	9-cis-epoxycarotenoid dioxygenase 3	HvNCED_fw	5'-CGCCCTCCATCCCTCCCATCTTCT-3'	132 bp
		HvNCED_rev	5'-CCGCCGCTAACTGTTTCCTCTTCC-3'	
HORVU3Hr1G007500	16.9 kDa class I heat shock protein 1	HvHsp17_fw	5'-TCGAGATCTCCGGCTGAATGC-3'	119 bp
		HvHsp17_rev	5'- CGGCAAGAACAACGACACAAC- 3'	
HORVU5Hr1G092160	Dehydrin 7	HvDhn1_fw	5'-GAGGAGGAAGAAGGGGATGAAG-3'	174 bp
		HvDhn1_rev	5'-AGCTTCTCCTTGATCTTGTCCA-3'	
HORVU.MOREX.r2.2HG0086370/ HORVU2Hr1G011070	Heavy metal-associated domain containing protein	HvFP1_fw	5'-CGCCGGGTGGCATACAAGAC-3'	148 bp
		HvFP1_rev	5'-CGGCGGAGGGGTCGCTCAT-3'	
	Cysteine protease	HvSAG39_fw	5'- CCCTGCCTAGCTTTCCTTCT-3'	168 bp

HORVU.MOREX.r2.5HG0424600		HvSAG39_rev	5′- GTGCGAGGATTAACGTGGAC-3′	
HORVU.MOREX.r2.2HG0139100	Subtilisin-like protease	HvSBT_fw	5′-CTCACTCTCTCCGCTCTCAC-3′	172 bp
		HvSBT_rev	5′-ATTTGGACGATGTATGGCCG-3′	
HORVU.MOREX.r2.4HG0316380	FAR1-related sequence 5	HvFRS5_fw	5′-CCTCTCGCTGTGACGGAACG-3′	131 bp
		HvFRS5_rev	5′-TCGGTGGAGGAACGGTGGG-3′	
HORVU.MOREX.r2.5HG0395730	Phytochromobilin:ferredoxin oxidoreductase	HvPFBS_fw	5'-TGGTATGGTCAAGCAAAGCA-3'	155 bp
		HvPFBS_rev	5'-GATTAGGCATTGCACACCCA-3'	
HORVU.MOREX.r2.4HG0302250	Acyl-CoA-binding domain-containing protein 4	HvACBD4_fw	5′-ACAGTAACTAGAGCAGGCCC-3′	162 bp
		HvACBD4_rev	5′-TGCAGCAACATGGTTGGATC-3′	
HORVU7Hr1G006630	Leucine-rich repeat receptor-like protein kinase family protein	HvLRR-RLK_fw	5′-CGAATACATACGGGGAGGCT-3′	161 bp
		HvLRR-RLK_rev	5′-AAGGAAGGAGTCATGCCCAA-3′	
HORVU.MOREX.r2.7HG0585880	F-box domain containing protein/zinc binding CCCH family protein	HvC3H12_fw	5'-CTCATGAGACCAGCACAGGA-3'	162 bp
		HvC3H12_rev	5'-CTAGCTTCTGTGGCCCTCTT-3'	
HORVU.MOREX.r2.5HG0352380	Myb/SANT-like DNA-binding domain protein	HvMSANTD_fw	5'-CATCTCCTCCCTCCATCCAC-3'	200 bp
		HvMSANTD_rev	5'-GTCCAGGCCTCTTTACTCCA-3'	
HORVU.MOREX.r2.4HG0305130	Auxin response factor	HvARF10_fw	5'-TGTTGAGCATGACTGGAGGA-3'	216 bp
		HvARF10_rev	5'-GAGACACGGTAGGGAAGGAG-3'	
